# Characterization and Generation of Male Courtship Song in *Cotesia congregata* (Hymenoptera: Braconidae)

**DOI:** 10.1371/journal.pone.0062051

**Published:** 2013-04-22

**Authors:** Justin P. Bredlau, Yasha J. Mohajer, Timothy M. Cameron, Karen M. Kester, Michael L. Fine

**Affiliations:** 1 Department of Biology, Virginia Commonwealth University, Richmond, Virginia, United States of America; 2 Department of Mechanical and Manufacturing Engineering, Miami University, Oxford, Ohio, United States of America; The Australian National University, Australia

## Abstract

**Background:**

Male parasitic wasps attract females with a courtship song produced by rapid wing fanning. Songs have been described for several parasitic wasp species; however, beyond association with wing fanning, the mechanism of sound generation has not been examined. We characterized the male courtship song of *Cotesia congregata* (Hymenoptera: Braconidae) and investigated the biomechanics of sound production.

**Methods and Principal Findings:**

Courtship songs were recorded using high-speed videography (2,000 fps) and audio recordings. The song consists of a long duration amplitude-modulated “buzz” followed by a series of pulsatile higher amplitude “boings,” each decaying into a terminal buzz followed by a short inter-boing pause while wings are stationary. Boings have higher amplitude and lower frequency than buzz components. The lower frequency of the boing sound is due to greater wing displacement. The power spectrum is a harmonic series dominated by wing repetition rate ∼220 Hz, but the sound waveform indicates a higher frequency resonance ∼5 kHz. Sound is not generated by the wings contacting each other, the substrate, or the abdomen. The abdomen is elevated during the first several wing cycles of the boing, but its position is unrelated to sound amplitude. Unlike most sounds generated by volume velocity, the boing is generated at the termination of the wing down stroke when displacement is maximal and wing velocity is zero. Calculation indicates a low Reynolds number of ∼1000.

**Conclusions and Significance:**

Acoustic pressure is proportional to velocity for typical sound sources. Our finding that the boing sound was generated at maximal wing displacement coincident with cessation of wing motion indicates that it is caused by acceleration of the wing tips, consistent with a dipole source. The low Reynolds number requires a high wing flap rate for flight and predisposes wings of small insects for sound production.

## Introduction

Insects display a wide range of acoustic signals used in species recognition and elicitation of courtship and mating [1]. Acoustic signals of several Hymenopterans have been described; perhaps the best characterized being part of the “waggle dance” of honeybees [2], [3], [4], [5], [6]. Parasitic wasps are highly diverse with over 50,000 described species across many families [7], and utilize wing movement or fanning to produce male courtship signals [8]. Wing fanning is hypothesized to draw female pheromones across the male’s body for orientation towards the female and to stimulate production of their courtship song [9]. As demonstrated by wing excision [10], [11], male courtship song in braconid wasps is caused by modulation of wing fanning. The song attenuates rapidly in air but also transmits through the substrate [12], [13], [10]. Acoustic signals have been characterized for the *Cotesia flavipes/sesamiae* (Cameron) species complex [14] and for a few other braconid wasps, including *Glyptapanteles flavicoxis* Marsh [15], *Aphidius ervi* Haliday [11], as well as five species of fruit fly parasitoids in the subfamily Opiinae [12], [16], [17]. Acoustic signals may play a role in reproductive isolation in the *Cotesia flavipes/sesamiae* species complex [14].

The gregarious endoparasitoid, *Cotesia congregata* Say (Hymenoptera: Braconidae), parasitizes the tobacco hornworm, *Manduca sexta* (L.) and other species of sphingid larvae that feed on a diverse array of plant families and species [18], [19]. This parasitoid serves as a component of a model system for host-parasitoid interactions and insect immunology [20], [21], insect learning [22], [23], [24], and tri-trophic interactions [25], [26]. Here we characterized the male courtship song of *C. congregata* and examined the mechanism of the high-amplitude “boing” component with high-speed videography and synchronized audio recordings. Based on general principles of sound generation [27], we expected that the fundamental frequency would match wing fanning rate, and the maximal sound amplitude would occur during maximal velocity of wing motion. The frequency matched the wing rate, as expected, but surprisingly the boing appears to be generated at the termination of the wing down stroke when displacement is maximal and wing velocity is zero.

## Materials and Methods

### Parasitic Wasps

Audio work utilized wasps from larvae of *M. sexta* collected in September and October 2010 from tobacco (*Nicotiana tabaccum* L.) at the Southern Piedmont Agricultural Research and Extension Center near Blackstone, Nottoway County, Virginia (37.0817, −77.9755). Wasps examined with high-speed videography came from a laboratory colony originating from collections at Blackstone, Virginia in 2005. Caterpillars, collected before parasitization status was known, were held in plastic containers (28×16×11 cm; 10–15 larvae in each) with tobacco leaves and then isolated into cups upon egression of parasitoids. Wasp cocoons were placed into individual clear gel capsules (size 00) 3–4 days after egression and resulting emergent adults were sexed under a dissecting microscope.

### Audio Recordings

To induce courtship signals, individual males from multiple cohorts were exposed to a female, immobilized in a drop of honey on a piece of tobacco leaf in an open plastic petri dish (14 cm diameter). Recordings were made in a sound isolation booth (Industrial Acoustics, Bronx, NY) at 23±1.5°C and 40–55% RH with a miniature omnidirectional microphone (DPA 4060, Longmont, CO; 20–20,000 Hz) held 2–3 mm from the male and a 702 High Resolution Digital Audio Recorder (Sound Devices, Reedsburg, WI; 48 kHz sampling rate, 24 bit resolution). Duration of signal components, fundamental frequency, and RMS sound pressure level re: 20 µPa (dB SPL) at 2–3 mm were determined using Raven Pro v1.3 (Cornell Lab of Ornithology, Ithaca, NY). All waveforms were high-passed filtered at 100 Hz, and frequency spectra were calculated for each signal component (Hann window, 3000 samples, 1.46 Hz resolution). Filtering resulted in a minor decrease (1 dB) in the sound pressure level at the fundamental frequency. Sound amplitude was calibrated by recording a 90 dB SPL 500 Hz test tone produced by a function generator (Tektronix CFG250, Beaverton, OR) through an audio monitor (Grass AM7, West Warwick, RI) with an SPL meter (Radio Shack, Fort Worth, TX). Since these sounds were recorded in the extreme near field, absolute levels should be considered approximations, but difference between the initial buzz, boing and terminal buzz should be reliable.

The initial buzz and first five distinct higher-amplitude sounds, termed “boings,” were analyzed for 21 individual wasps, and data were averaged so that each wasp was treated as an *N* of 1. Differences in fundamental frequency and amplitude among the initial buzz, boing, and terminal buzz elements were compared using a repeated measures analysis of variance (ANOVA) followed by Tukey’s test with Prism 5 (GraphPad Software, San Diego, CA), and correlation between selected elements was determined using linear regression with R v2.12.2 (R Foundation for Statistical Computing, Vienna, Austria).

### Video Recordings

High-speed video images of courting males were recorded using a FASTCAM PCI R2 (Photron, San Diego, CA) camera at 2,000 frames per second (0.5 ms per frame) with a resolution of 256×120 pixels through a TV zoom lens (NAVITAR, Rochester, NY) on macro (12.5–75 mm focal length, f/1.8 aperture). Males were exposed to an immobilized female in honey in a clear plastic box (2×2×1 cm) with no lid. The camera was positioned 4 cm above the wasp, and light was provided by a 150 W fiber optic ring microscope light (Schott, Elmsford, NY) attached to the end of the camera. Wasps were repositioned with forceps to capture video at different angles. Simultaneous audio recordings were made with an Etymotic (Elk Grove Village, IL) ER-7C probe tube microphone (+20 dB amplification) connected to the camera software. The video camera was synchronized with sound through a triggerbox (NI BNC-2110, National Instruments, Austin, TX). The tip of the probe tube was 1 cm from the wasp at a slight angle from above. The greater recording distance (1 cm vs 2–3 mm) was necessary to avoid obscuring the visual field of the camera. Space constraints prohibited our use of a velocity microphone, which would have obscured the camera, during high-speed recordings. Due to the increased noise produced by the camera and light and lower sensitivity of the microphone, these recordings had a higher noise level, but the waveform of the high amplitude boing element was clear. Sound was recorded at a sampling rate of 40 kHz so that sound records provided greater resolution than video recordings. At a distance of 1 cm, the difference between the propagation of sound, 0.03 ms, and light was not considered in the analysis. Camera speed was confirmed by connecting the synch out port of the camera to an oscilloscope, and relative synchronization of the audio and video outputs was determined by filming the collision of the metal tip of a miniature modal analysis hammer (PCB Piezotronics, Depew, NY) against a metal air table. Within the 0.5 ms resolution of the camera the outputs were synchronized.

Video and audio were analyzed using Photron Motion Tools, Photron FASTCAM Viewer, and the BioPac System (Acknowledge software 3.7, Goleta, CA). Nineteen 8-second videos were recorded from fourteen individual males at a variety of positions (lateral, dorsal, ventral, anterior, and posterior) to observe patterns of movement. Motion was qualitatively similar in all 14 wasps. Frame-by-frame video analysis of three boings from six individuals was performed to determine if the fundamental frequency corresponds to wing beat rate. We performed a more detailed frame-by-frame analysis of six boings, two each from three individuals to determine the relationship of sound waveform to wing and abdomen motion. Amplitude, down-stroke displacement in degrees, velocity, displacement from the vertical, and abdominal angle from the horizontal for each of multiple wing strokes producing the boing were measured. Data from the two replicates were averaged and compared across the three males (n = 3).

## Results

### Audio

**Figure 1:**
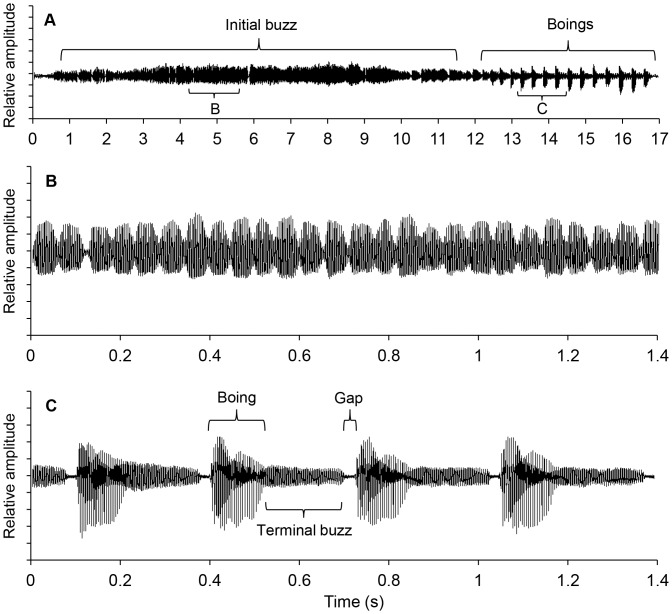
Oscillograph of typical male courtship song of *Cotesia congregata* with a buzz followed by boings. (A) Complete song. (B) Expanded selection of initial buzz. (C) Expanded selection of four boings illustrating the initial high amplitude component followed by a lower amplitude terminal buzz and short gap.

The courtship song of *C. congregata* consisted of a two part signal (Audio S1), initiated when males were within 2–3 cm of a female. The song began with a sequence of amplitude-modulated buzz sounds produced by wing fanning that lasted up to a minute (mean ± SE: 14.0±1.4 s; Fig. 1). Song sequences ended with a series of pulsatile-sounding higher amplitude boing sounds (17±1 boings per song; range 9–27), which typically occurred when the males were within 1 cm of a female, i.e., closer than for initial fanning. Males were usually stationary during boing production. The angle between the abdomen and thorax increased during initial wing strokes of a boing and then decreased (see *Video: motion and sound* section). Males continued to produce boings until they attempted to mate with the female. Other observations of males displaying to free females indicate that males either attempt to mate at this point or the female moves away.

**Figure 2:**
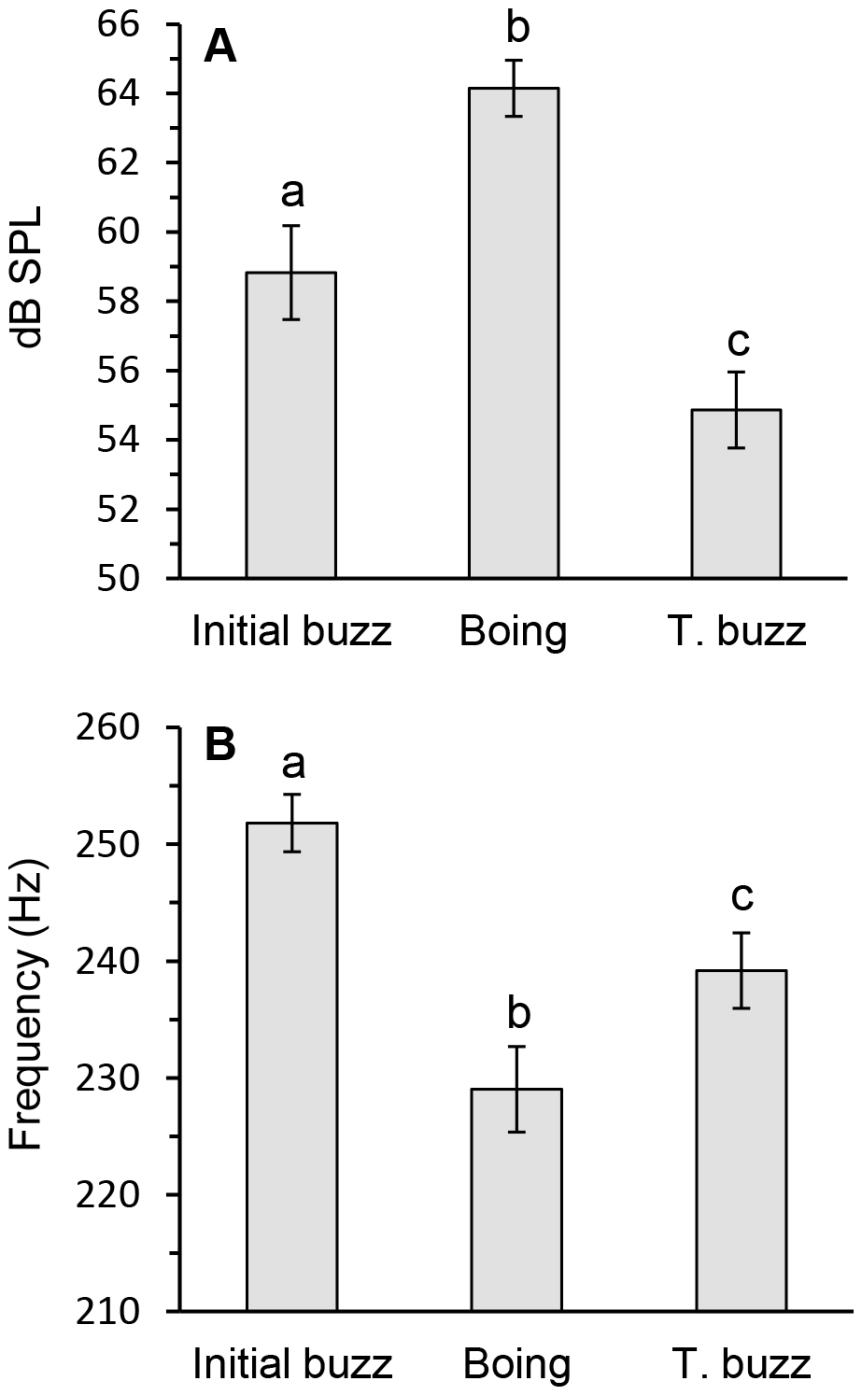
Sound pressure level and fundamental frequency of the male courtship song of *Cotesia congregata*. (A) Sound pressure level re: 20 µPa at 2–3 mm (dB; mean ± SE) and (B) fundamental frequency (Hz; mean ± SE) of initial pre-boing buzz, boing, and terminal buzz components. Different letters indicate significant differences (p<0.01). *N* = 21 wasps.

Each boing consisted of an initial rapid onset high-amplitude portion that decayed into a lower amplitude terminal buzz. The buzz was then followed by a brief silent gap when wing movement stopped (Fig. 1). Amplitude increased from 58.8±1.4 dB during the initial buzz to 64.2±0.8 dB during the boing and then decreased to 54.9±1.1 dB during the terminal buzz (repeated measures ANOVA, F_2, 40_ = 78.8, p<0.0001; Fig. 2A). Transitions between the boing and terminal buzz were variable with some waveforms having a steep and others a more gradual transition. Duration of the boing, terminal buzz and silent period were 121.2±2.8, 203.6±6.4, 22.8±0.7 ms respectively. Boing and buzz durations varied independently (r^2^ = 0.068, p = 0.134); signal length increased with terminal buzz duration (r^2^ = 0.779, p<0.0001) but not boing duration (r^2^ = 0.008, p = 0.695). Thus, overall signal length was more dependent on the terminal buzz than the boings. Boing and gap duration were negatively correlated (r^2^ = 0.185, p = 0.033).

**Figure 3:**
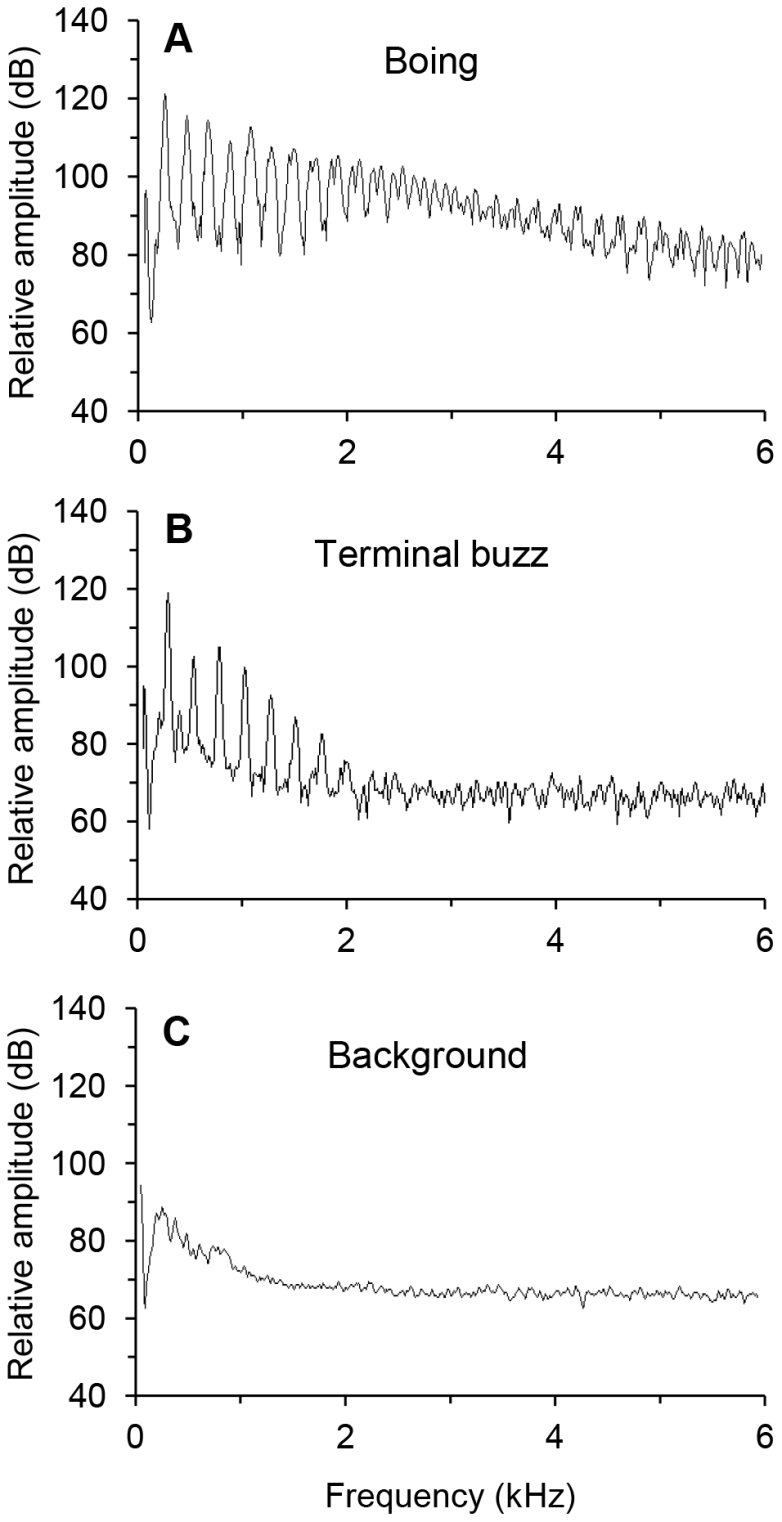
Frequency spectra of components of the male courtship song of *Cotesia congregata*. (A) Boing, (B) terminal buzz, and (C) background noise.

Fundamental frequency of the song was generated by the wing fanning rate (see *Video: motion and sound* section), which produced a harmonic series that decreased with frequency (Fig. 3). Fundamental frequency differed between initial buzz, boing, and terminal buzz components (F_2, 40_ = 34.8, p<0.0001; Fig. 2B): the mean initial buzz was 252±3 Hz, the boing was 229±3 Hz, and the terminal buzz was 239±3 Hz. The decrease in fundamental frequency during the boing coincided with larger wing strokes (*Video: motion and sound* section). The boing frequency spectrum decreased about 40 dB from its fundamental frequency with energy above background at 7 kHz, whereas the weaker terminal buzz decreased ∼50 dB before reaching background levels at ∼2 kHz (Fig. 3).

### Video: Motion and Sound

**Figure 4:**
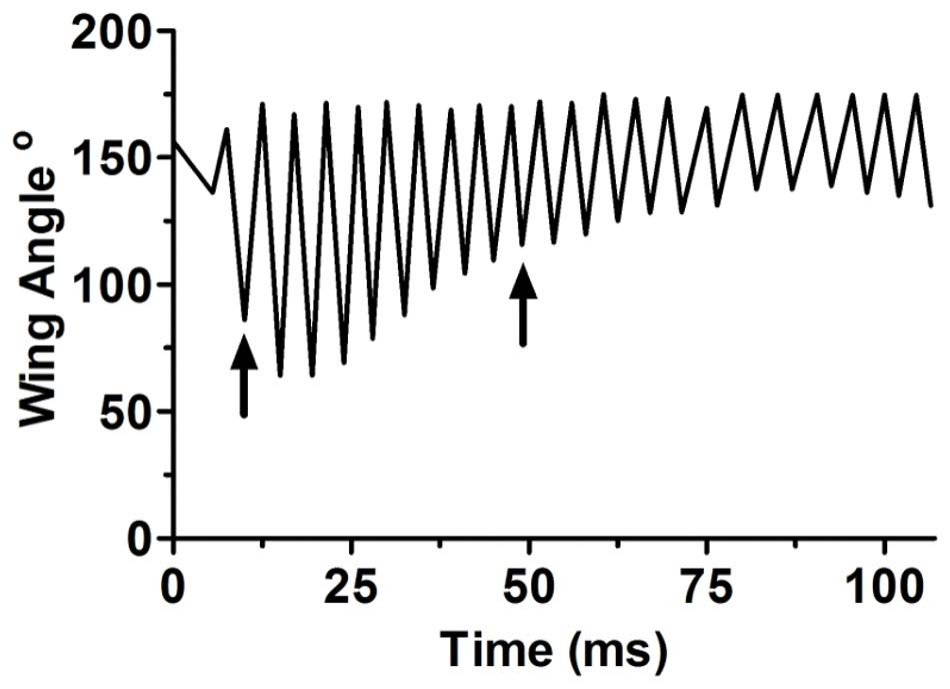
Change in wing angle over time during a single boing. Vertical wing angle at the beginning and end of successive wing strokes during a typical boing (vertical plane toward the substrate = 0°) of the male courtship song of *Cotesia congregata*. The first arrow indicates the first wing stroke producing audible sound and the second arrow indicates the downstroke producing the highest amplitude sound.

**Figure 5:**
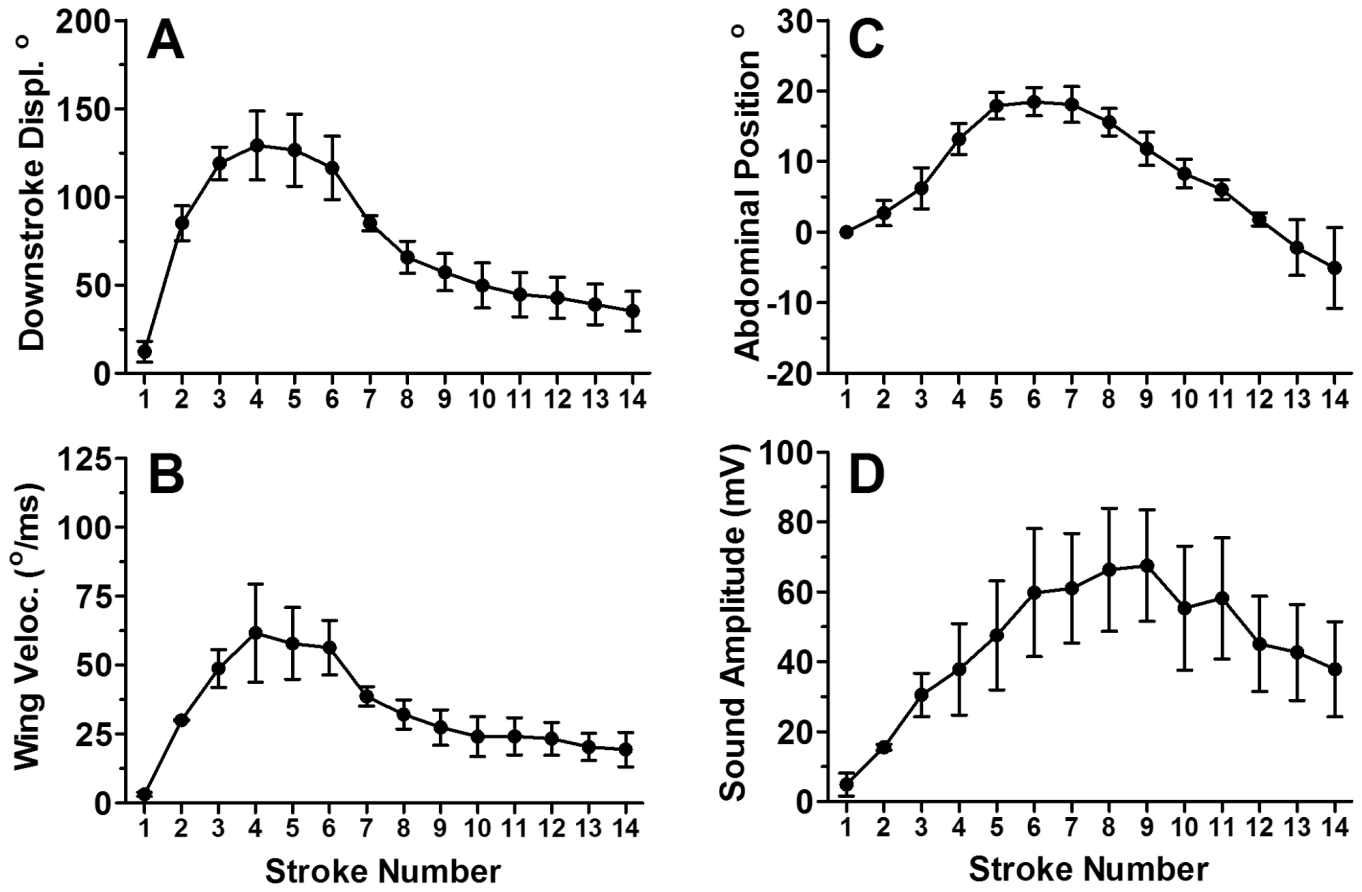
Measurements of wasp movement and sound amplitude of each wing stroke during a single boing. (A) Wing downstroke displacement (mean ± SE), (B) maximum downstroke velocity, (C) abdominal angle (horizontal = 0°), and (D) sound amplitude during successive wing strokes during a boing averaged from frame by frame analysis of the courtship song produced by three males of *Cotesia congregata*. Strokes from boings with more than 14 pulses were deleted so that *N* was always 3.

During pre-boing fanning, the wings moved downward from a dorsal position to ∼45° angle above the horizontal before returning. The silent gap before each boing was visible as the wings paused in the dorsal position before initiating a series of large amplitude motions. Boings began with a small silent down stroke followed by several more strokes of increasing displacement to the fourth stroke (Videos S1, S2). Sound was evident by the second or third stroke (Fig. 4) and after the fourth stroke, displacement decreased slowly and then more rapidly (Fig. 5A). At maximal excursion the wings dropped about 120° from the starting position and were below the horizontal plane. The two pairs of wings did not touch each other during the upstroke nor did they hit the abdomen or substrate during the down stroke. Wing velocity (°/ms) paralleled displacement (Fig. 5B). Wing cycle duration averaged 4.6±0.1 ms, and there were 10–15 wing-beat cycles before the boing transitioned to the terminal buzz. Wing beat frequency determined from video recordings of wing strokes from six wasps averaged 216±5 Hz, comparable to the fundamental frequency in audio recordings (Fig. 2B). Abdomen elevation increased to 18.4±2.0° above the horizontal by the fourth or fifth stroke, maintained the elevation for several strokes, and then decreased in a linear fashion (Fig. 5C). Sound amplitude increased for eight to nine wing strokes and then decreased as the boing component decayed into the lower amplitude terminal buzz (Fig. 5D). The mean terminal buzz utilized 53±2 wing beats, with each stroke averaging 4.3±0.04 ms. As wing beat amplitude decreased during the terminal buzz, stroke frequency increased to 234±2 Hz, similar to the value found in audio recordings (Fig. 2B).

**Figure 6:**
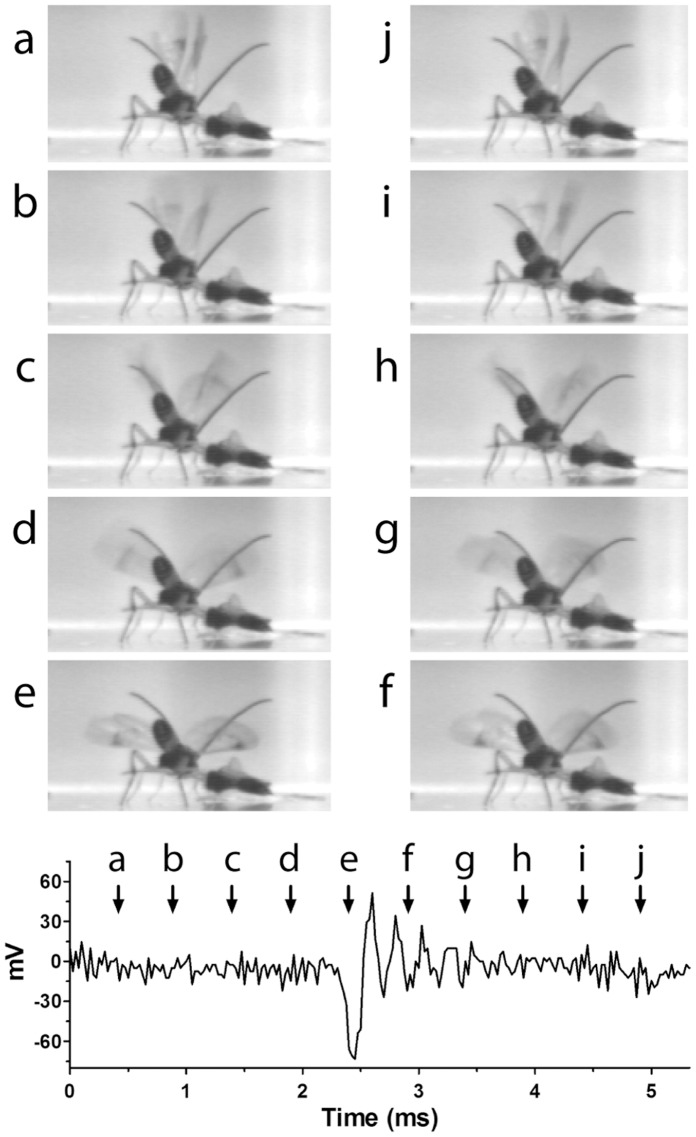
Images of a single wing stroke during a boing matched to sound amplitude. **Above:** High-speed camera photographs (2,000 fps) of one wing cycle during a boing produced by downward (a–e) and upward (f–j) wing movement from a male *Cotesia congregata* displaying to an immobilized female. Each image represents 0.5 ms. Note that wings are less clear in the middle of the down and upsweep (images b–d and g–i) due to rapid movement. **Below:** Oscillograph of one cycle of a boing with wing positions in a-j keyed to time of occurrence.

Observation of visual and audio recordings indicated that the boing sound was produced at the end of the wing downstroke when wing displacement was maximal and its velocity was zero (Fig. 6), supporting the absence of a direct proportionality between wing velocity and sound amplitude. Within a single wing cycle the sound waveform began with a negative peak corresponding to wing depression. The first cycle of the waveform averaged 0.32±0.01 ms, and subsequent cycles exhibited an exponential decay and a period of 0.22±0.01 ms. The first sound cycle appeared to be a combination of forced and resonant responses from acceleration of the wing tip when wing velocity ceased. Subsequent cycles are likely dominated by wing resonance of ∼5 kHz (a period of ∼0.2 ms), whereas the 210–220 Hz wing beat frequency is a purely forced response.

**Figure 7:**
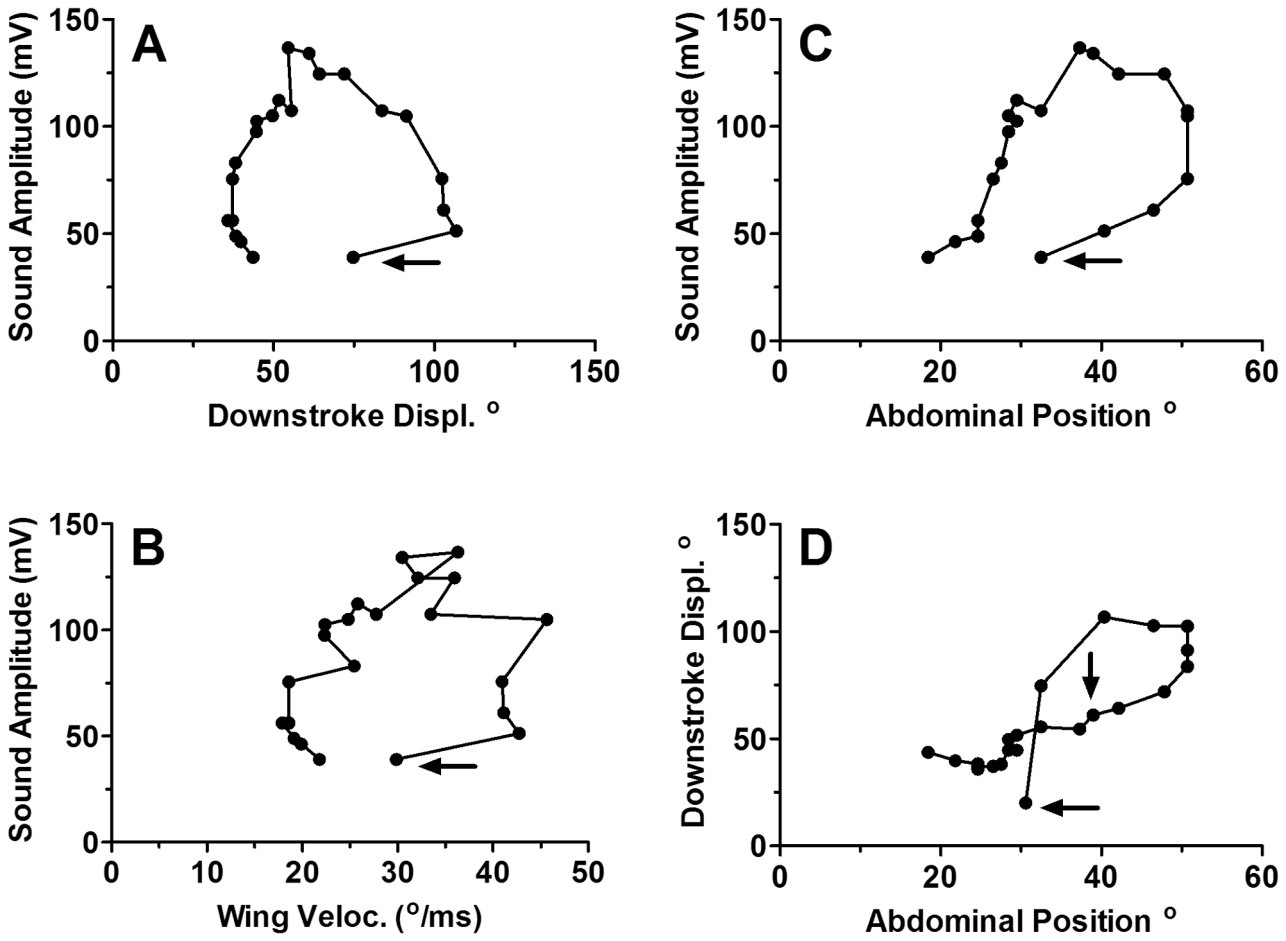
Relationship of sound amplitude to measurements of wasp wing and body movement. (A) Down-stroke displacement, (B) wing angular velocity, (C) abdominal angle related to sound amplitude, and (D) down-stroke displacement to abdominal position during consecutive wing strokes of a single boing in the male courtship song of *Cotesia congregata.* The horizontal arrows mark the first wing stroke; the vertical arrow in (D) marks the stroke producing the greatest sound amplitude. Hysteresis in the figures indicates that although wing velocity and abdomen elevation increase with sound amplitude at the beginning and decrease at the end of a boing, they are not causative.

Because wing movement and abdomen elevation tended to parallel changes in sound amplitude during the boing (Fig. 5), their role in sound generation was unclear. Plots of wing displacement, wing velocity, and abdominal position against amplitude and abdominal position against displacement from individual boings indicated hysteresis between these parameters during the course of the boing (Fig. 7). Analyses of replicate boings from the same wasp yielded similar results. There was qualitative variation among the three wasps, but the basic pattern was invariant. Over a two-fold variation in wing displacement, velocity and abdominal angles were associated with the same sound amplitude (Fig. 7A, B, C), and all exhibited a counter-clockwise hysteresis pattern although hysteresis in the relationship of displacement to abdominal position was clockwise (Fig. 7D). We therefore conclude that wing and abdominal movements changed with time as did sound amplitude, but neither explained the timing of the high amplitude sound cycles and were therefore not directly causative.

## Discussion

The courtship song of *Cotesia congregata* consists of a two-part signal that begins with a long amplitude-modulated buzz (wing fanning) and concludes with a series of higher amplitude boings, each of which decays into a terminal buzz. The three components differ in amplitude and frequency, which were inversely related; i.e. when amplitude increased, frequency decreased. The higher amplitude boing requires greater wing excursion and therefore wing strokes of longer duration, thereby generating a lower fundamental frequency. The initial fanning may also serve to localize the female pheromone source [9], and the number and amplitude of boings likely reflects an honest signal of male quality for female mate choice. In other parasitic wasps studied, wing fanning produces sound frequencies in harmonic series at multiples of the fundamental frequency, which vary between 122 and 314 Hz across species [15], [12], [16], [11], [17].

Abdomen elevation during high-amplitude components has not been described in the literature for other parasitic wasps. This movement may not be involved directly in sound production because the wings do not strike the abdomen during the downward sweep. Its function is unclear because abdominal muscles are typically separate from thoracic muscles [28], and maximum sound amplitude lags behind abdominal movement (Fig. 5, 7C). Possibly, abdominal elevation could reposition the body for balance or facilitate vibrations transmitted to the substrate, as reported for some species of lacewings [29] and stoneflies [30]. Alternatively, abdomen elevation may be associated with release of a chemical signal. Abdomen elevation is unlikely to provide a visual mating signal because the female typically faces away from the male during courtship.

The sound probe tube microphone was in the near-field for sound radiation, so the sound measurements may be highly sensitive to slight variations in position due to local reflections and reactive sound components. High sensitivity to position for the amplitude and phase measurements of the sound would be indicated by a high variation among the measurements. However the low variation and consistency of the amplitude and phase measurements (Fig. 5) indicates that reactive effects were small. The recordings were aided by experimental conditions that ensured the male maintained a stationary position during boing production. Amplitude indicates that acoustic signals in air would be effective over short distances. Based on spherical spreading, a 64 dB SPL signal recorded at 2–3 mm would be equivalent to a source level of only about 10 dB at 1 m. Using playback from a speaker in air that evoked no measurable substrate vibrations, Danci et al. [15] demonstrated that female parasitic wasps were attracted to the male’s airborne song. This result does not rule out a contribution from leaf vibrations under natural conditions.

Of the 15,000 described species in the Braconidae [31], courtship sounds have been described for eleven other species, representing nine genera in three subfamilies. Compared to other braconids, *C. congregata* has a relatively complex song that consists of two major parts, the initial buzz and the boings, with each boing followed by a terminal buzz. For example, *C. marginiventris,* the closest relative to *C. congregata* [32] for which courtship songs have been described, produces structurally distinct songs with shorter repeated pulses that decay into a warble [12]. Other braconids produce signals that range in structure and complexity: *Glyptapanteles flavicoxis* utilizes amplitude-modulated wing fanning [15], members of the subfamily Opiinae produce a series of repeating pulses [12], [16], [17], and males of the *Cotesia flavipes/sesamiae* complex produce separate repeating buzz and pulse components [14].

The wing-beat frequency during courtship displays has been examined photographically [33] and acoustically [17] for the braconid, *Psyttalia concolor*; however, these studies did not relate wing movement directly to sound generation. For typical wing sound production, including *Drosophila* spp., acoustic pressure is proportional to velocity [34]. Our finding that the boing sound was generated at maximal wing displacement when velocity is zero was initially surprising, but we believe justified. Sampling theory can be applied to reconstruct the full wing-flap position versus time profile as long as more than two frames are obtained for each wing flap. The high-speed video captured ∼10 frames for each full wing-flap cycle, exceeding the sampling theory requirement. This allows us to correlate the wing position with the sound even though the video and sound sampling rates were different. Note however that camera data are not useful in explaining the waveform of the sound signal, which appears to result from a forced response caused by wing repetition rate (∼210–229 Hz) and wing resonance (∼5 kHz). Because sound cannot be generated by a static structure, we considered several hypotheses to explain sound generation.

**Figure 8:**
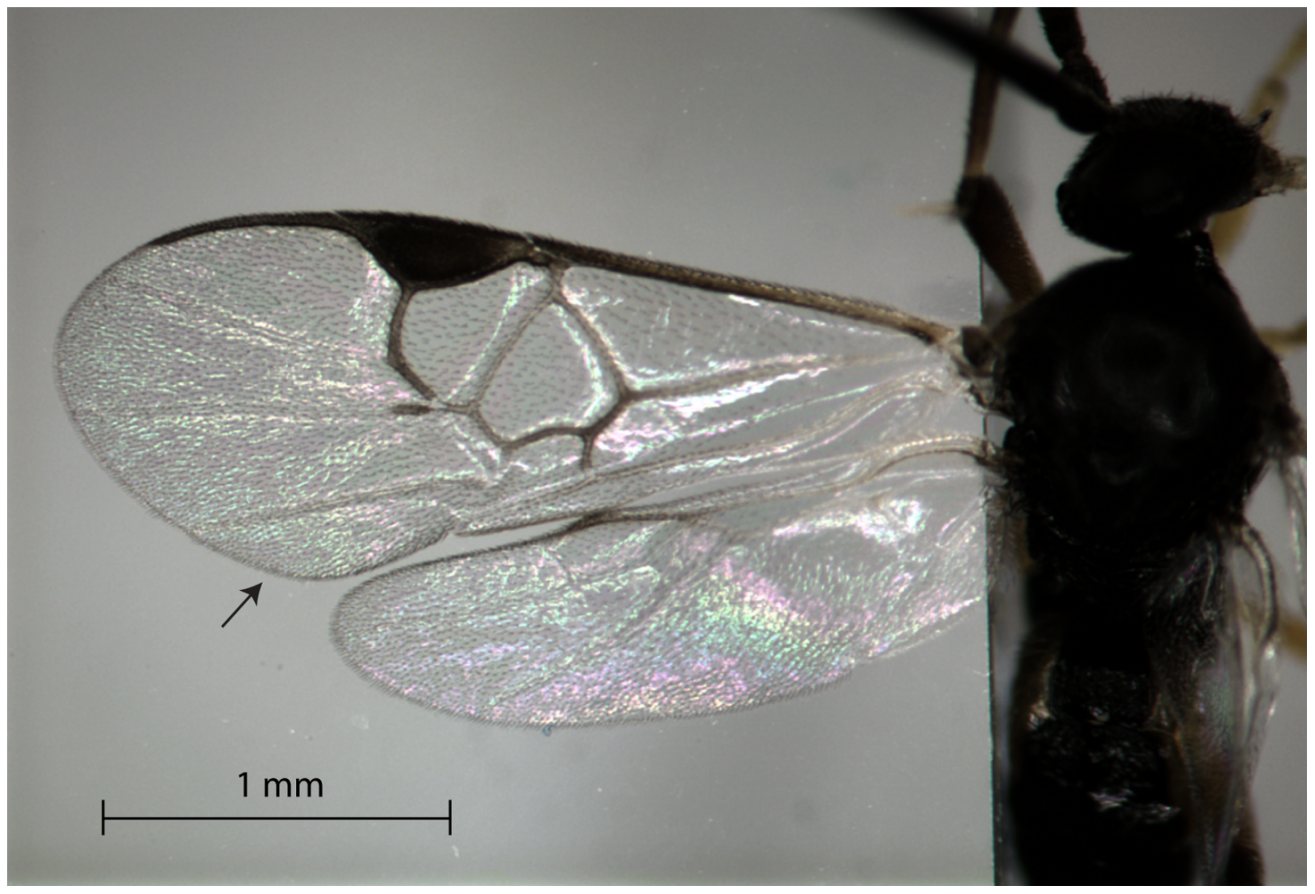
Dorsal view of one pair of wings of a male *Cotesia congregata*. The wings are supported by a microscope slide (vertical line near the wing base). The terminal fold used in calculations of wing speed is indicated by the arrow.

The physics of bullwhips, which generate a snap by tip acceleration to supersonic speeds (340 m/s), has received considerable attention from physicists [35] and has been proposed as a possible mechanism of sound generation for the tail tip of sauropod dinosaurs [36] and wing snaps in manakin birds [37]. As in many hymenopterans, the paired wings of *C. congregata* are hooked together by hamuli and function as a unit; wings never touch the abdomen, the substrate, or the opposite wings ruling out wing collisions as the sound source. The larger forewings are small (2.38 mm long), and veins that support the wing terminate before the wing tip where there is a series of four less-supported folds in the distal wing (Fig. 8) that could be subject to a whip-like motion during rapid wing deceleration. Using values measured in this study, we calculated wing-tip speed with the equation:
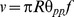
where *v* is the peak velocity of the wing tip, *R* is wing length (2.38e−3 m), θ*_pp_* is wing peak-to-peak angle (2π/3 rad/cycle), and *f* is wing-beat frequency (230 Hz). Wing velocity would be 32.4 m/s, which is about 10% of the speed of sound. Making the extreme assumption that all energy in the wing is transferred to the posterior terminal wing fold (Fig. 8), about 11.4% of area (measured with grid squares), increases speed to 123 m/s, about 36% of the speed of sound. We therefore reject the whip-snap hypothesis and suggest that it has limited applicability to other biological systems.

Previous work on the directionality of the sounds produced by dancing honeybees indicates that the up and down wing motion has the directionality of a dipole source: a bidirectional sound field that decays rapidly in air [38]. There is no net introduction of fluid by a dipole (cancellation of up and down wing motion), and only net force on a fluid causes energy to be radiated as sound [39]. Equations of pressure of a dipole are typically related to velocity [39]. However, because pressure lags velocity by 90°, it will be in phase with acceleration. The precise timing relationship in the parasitoid wing-sound generation suggests an almost pure dipole source, although we do not rule out an aerodynamic component due to interacting vortices produced by the leaking of higher pressure under the wings to the lower pressure above. Sounds generated from vortices produced by flapping wings have been demonstrated in blowflies [40] and modeled during bumblebee flight [41]. Our measurement of the phase relationship between dipole wing acceleration and sound pressure may be the first demonstration in biological sound generation.

The dipole acceleration model best explains our finding that boing amplitude increases over several cycles as wing displacement decreases. The indirect flight muscles of many insects are excited by a combination of neural input and stretch-induced activation [42], [43]. Toward the end of a large downstroke displacement, when the antagonist elevator muscle is activated, the wings likely slow down, decreasing acceleration at the end of the excursion. Changing direction in midstroke would reverse movement during a faster phase of contraction (higher instantaneous velocity) and therefore increase acceleration and sound amplitude even with a smaller wing stroke because velocity is proportional to *A*ω and acceleration to *A*ω^2^, where *A* is displacement and ω is 2π*f*. These relationships explain our finding that two fold changes in wing velocity failed to affect sound amplitude in the hysteresis measurements.

In order to understand the relationship of wing beat frequency to sound generation in *Cotesia*, we calculated the Reynolds number for a 3-dimensional flapping wing, *Re_f_*_3_, following Shyy et al. [44] using the equation:
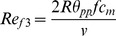
where 2*R*θ*_pp_f* is the mean angular velocity of the wing tip (reference velocity), *c_m_* (1.07e−3 m) is the mean wing chord length (reference length), and ν is the kinematic viscosity of air (1.51e−5 m^2^/s at 20°C). The Reynolds number is approximately 1000 (959 for 216 Hz and 1020 for 230 Hz wing rate). This compares to ∼100 for *Drosophila melanogaster* [45] and 18 for the tiny egg parasitoid *Encarsia formosa*, which has smaller wings [46]. Larger Hymenopterans such as honeybees and bumblebees have Reynolds numbers that range from 1000 to 8800 [46], [41]. As for other small insects with relatively low Reynolds numbers, *C. congregata* may implement a “clap and fling” mechanism for flight in which the wings come together and then fling apart while the trailing edge remains momentarily in contact [46], [47], [45]. Wing fanning during courtship does not utilize the clap and fling mechanism because the wings do not contact nor do the trailing edges stay together (Video S1). This difference may allow the wasp to remain on the substrate during courtship by not generating enough lift for flight. The low Reynolds number in *C. congregata* indicates a poor lift to drag ratio, therefore small insects such as parasitic wasps and vinegar flies require a high wing flap rate to generate adequate lift for flight, which predisposes them to produce sound that can later be selected for communication.

## Supporting Information

Audio S1 **Audio recording of courtship song of a male** ***Cotesia congregata*** **.** The song starts with wing fanning producing a buzz component, followed by a series of 22 boings.
(WAV)

Video S1 **High-speed video of the courtship song of a male** ***Cotesia congregata*** **(posterior view).** The video is one sixth of a second of a display of a boing filmed at 2000 frames per second slowed to 20 frames per second. The male wasp is displaying to an immobilized female. The video starts with the end of a terminal buzz preceding the pause before the next boing. Each boing starts with the wings elevated in an almost vertical position preceded by a silent period with no wing motion. The boing transitions to a terminal buzz with less wing displacement.
(AVI)

Video S2 **High-speed video of the courtship song of a male** ***Cotesia congregata*** **(lateral view).** The video is one sixth of a second of a display of a boing filmed at 2000 frames per second slowed to 20 frames per second. The male wasp is displaying to an immobilized female. The video starts with the end of a terminal buzz preceding the pause before the next boing. Each boing starts with the wings elevated in an almost vertical position preceded by a silent period with no wing motion. Note the abdomen elevation during the high amplitude wing displacements followed by multiple lower displacement wing cycles during the terminal buzz.
(AVI)
